# Neural circuit mechanisms of sensorimotor disability in cancer treatment

**DOI:** 10.1073/pnas.2100428118

**Published:** 2021-12-15

**Authors:** Stephen N. Housley, Paul Nardelli, Travis M. Rotterman, Timothy C. Cope

**Affiliations:** ^a^School of Biological Sciences, Georgia Institute of Technology, Atlanta, GA 30332;; ^b^Integrated Cancer Research Center, Georgia Institute of Technology, Atlanta, GA 30309;; ^c^Parker H. Petit Institute for Bioengineering and Bioscience, Georgia Institute of Technology, Atlanta, GA 30309;; ^d^Wallace H. Coulter Department of Biomedical Engineering, Emory University and Georgia Institute of Technology, Atlanta, GA 30332

**Keywords:** sensory encoding, synaptic function, spinal circuits, sensorimotor disability, cancer treatment

## Abstract

Cancer survivors rank sensorimotor disability among the most distressing, long-term consequences of chemotherapy. Disorders in gait, balance, and skilled movements are commonly assigned to chemotoxic damage of peripheral sensory neurons without consideration of the deterministic role played by the neural circuits that translate sensory information into movement. This oversight precludes sufficient, mechanistic understanding and contributes to the absence of effective treatment for reversing chemotherapy-induced disability. We rectified this omission through the use of a combination of electrophysiology, behavior, and modeling to study the operation of a spinal sensorimotor circuit in vivo in a rat model of chronic, oxaliplatin (chemotherapy)–induced neuropathy (cOIN). Key sequential events were studied in the encoding of propriosensory information and its circuit translation into the synaptic potentials produced in motoneurons. In cOIN rats, multiple classes of propriosensory neurons expressed defective firing that reduced accurate sensory representation of muscle mechanical responses to stretch. Accuracy degraded further in the translation of propriosensory signals into synaptic potentials as a result of defective mechanisms residing inside the spinal cord. These sequential, peripheral, and central defects compounded to drive the sensorimotor circuit into a functional collapse that was consequential in predicting the significant errors in propriosensory-guided movement behaviors demonstrated here in our rat model and reported for people with cOIN. We conclude that sensorimotor disability induced by cancer treatment emerges from the joint expression of independent defects occurring in both peripheral and central elements of sensorimotor circuits.

## Significance

Severe and persistent disability often undermines the life-saving benefits of cancer treatment. Pain and fatigue, together with sensory, motor, and cognitive disorders, are chief among the constellation of side effects that occur with the platinum-based anticancer agents used in a majority of cancer treatments worldwide. These disabilities remain clinically unmitigated and empirically unexplained as research concentrates on peripheral degeneration of sensory neurons while understating the possible involvement of neural processes within the central nervous system. The present findings demonstrate functional defects in the fundamental properties of information processing localized within the central nervous system. We conclude that long-lasting sensorimotor and possibly other disabilities induced by cancer treatment result from independent neural defects compounded across both peripheral and central nervous systems.

Neurotoxic effects weigh against the benefits of chemotherapy in treating cancer. Pain, sensory abnormalities, fatigue, and impaired mobility and cognition, collectively referred to as chemotherapy-induced neurotoxicity, diminishes quality of life and limits functional capacity in nearly all patients (1–4). These effects are expressed with platinum-based agents, including oxaliplatin, which are used in 50% of chemotherapy cases worldwide (5, 6). The debilitating side effects of platinum-based therapy are an active area of investigation in multiple clinical and scientific domains, including cancer biology, sensorimotor and cognitive neurobiology, rehabilitation, oncology, and neurology. Thus far, these efforts have fallen short of delivering preventative measures and only a single treatment achieves limited efficacy (9).

Among the chief complaints, patients report deficits in gait, balance, skilled movements, and other sensory-guided activities. These features of reduced mobility can variously persist months and even years into disease-free survival (7, 8). The lack of effective prevention or treatment for these chronic sensorimotor deficits emphasizes the poor understanding of the underlying pathophysiology.

Peripheral sensory neuropathy provides the most accessible explanation for sensorimotor disability in chemotherapy-induced neuropathy. Research efforts concentrate on treating physical degeneration of sensory peripheral axons in an attempt to restore the mechanosensory modalities (e.g., touch and proprioceptive senses), which are essential for guiding movement. However, the sufficiency of peripheral sensory degeneration as the sole or even primary explanation for sensorimotor impairment remains untested, and clinical studies report inconsistency in the relationship between disability and peripheral neuropathy (10). If not by structural neuropathy, then sensorimotor disability might arise from defective sensory encoding expressed by the substantial number of sensory neurons that do not degenerate (10, 11). The question remains, however, whether reversing neuropathy, either structural or functional, will prove sufficient to restore normal movement. Although indispensable, sensory input is only one element contributing to the integrated function of neural circuits that ultimately determine behavior and its disorders. We recently reported that motoneurons, the output neuron common to all sensorimotor circuits, exhibit sporadic firing attributable to intrinsic changes in excitability observed in our rat model of chronic oxaliplatin-induced neuropathy (cOIN) (12). With respect to integrative processes that translate circuit input into output, nothing is known, but possible involvement is supported by growing evidence for chemotherapy-induced changes occurring within the central nervous system (12–14). In the present study, we aimed to evaluate dysfunction in a more complete set of sensorimotor circuit elements, every one of which would require restoration in order to effectively reverse sensorimotor disability.

The system of neural circuits most relevant to sensorimotor disability in cOIN is one that utilizes mechanosensory information to correct for perturbations and errors in intended movements. Embedded within this system is a spinal sensorimotor circuit that translates information from multiple muscle and cutaneous propriosensors into short-latency muscular responses that oppose perturbation of intended movements. Through this circuit, propriosensory information encoded in the firing of sensory neurons supplying muscle spindles and tendon organs is integrated both monosynaptically and polysynaptically by premotor interneurons to coordinate the activity of motoneurons and muscles (15, 16). Spinal sensorimotor circuit dysfunction is likely responsible for reducing deep tendon reflexes and alterations in gait patterns in patients with cOIN (17) and possibly also for explaining mistakes in limb placement observed in patients following cancer treatment (18) and in our rat model (20). In both cases, peripheral neuropathy, physical or functional, is a likely contributor (19) but in neither case has there been any examination of processes that translate sensory information through the whole spinal sensorimotor circuit. The sufficiency of impaired sensory information alone to explain disability remains untested, in part because there has been no assessment of either the magnitude or specific content of propriosensory information lost to neuropathy. Nor is it known whether central elements of the spinal sensorimotor circuit adapt in ways that either compensate or further exacerbate the consequences of sensory deficiency.

The present preclinical study of cOIN was designed to examine a spinal sensorimotor circuit for defects in key neural functions, including propriosensory encoding in the periphery and integration within the central nervous system. We found that propriosensory encoding of a muscle’s biomechanical responses to stretch, although partially preserved, exhibited stereotypical defects shared by multiple classes of muscle propriosensors. Defective propriosensory encoding alone was not sufficient, however, to explain the additional, severe reduction of synaptic potentials produced through premotor circuits converging on motoneurons. Finally, we demonstrated correspondence between these sensorimotor circuit defects and specific kinematic errors in a sensorimotor task performed by rats with cOIN. We conclude that movement disabilities induced by chemotherapy for cancer emerge from independent defects compounded across not only peripheral but also the central nervous system, wherein we find damage to fundamental neural processes, including excitability and synaptic communication.

## Results

### Cancer Treatment Impairs All Types of Muscle Propriosensors.

Our previous study identified significant, functional encoding deficiencies caused by cancer treatment in one specific class of propriosensory neuron, namely the Ia muscle–spindle neuron (20). Although related to encoding defects in Ia neurons, the long-term sensorimotor disorders we find following cancer treatment may also depend on impaired function of additional, propriosensory cell types that encode diverse, biomechanical features. In order to test this possibility, we performed in vivo electrophysiological studies of type I unclassified spindle (I_un_), Ib (Golgi tendon organs), and II propriosensory neurons that variously encode muscle dynamics [i.e., unique static and time-varying parameters of muscle biomechanics measured as muscle force, position, velocity, and stiffness (21)]. We chose to represent muscle dynamics with the viscoelastic restoring force produced by the nonreflexive muscle. Muscle force is the most physiologically relevant variable encoded by Ib neurons, and it is shown to provide the best fit to the firing profile of Ia neurons under these experimental conditions (22).

**Fig. 1.**
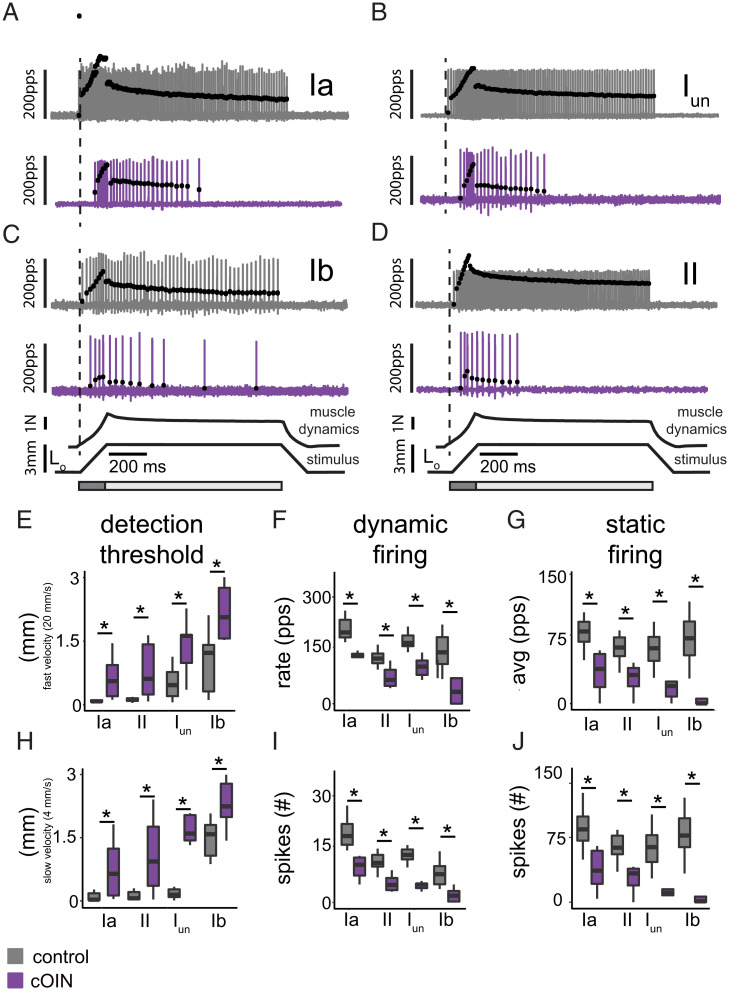
Chemotherapy for cancer impairs all muscle propriosensory neurons. Representative cases of spiking activity in control (gray) and cOIN (purple) as a measure of sensory encoding in Ia (*A*), I_un_ (*B*), Ib (*C*), and II (*D*) propriosensors. Black circles plot instantaneous firing rates (pulses per second [pps]) of corresponding spike (action potential) intervals. Dashed vertical lines mark onset of movement (3 mm from resting length: L_o_) shown in bottom trace divided into dynamic and static phases, respectively, by dark gray (150-ms duration after stretch command onset) and light gray (1-s duration after the dynamic phase) bars. Traces below indicate imposed muscle movement (stimulus) and muscle response (muscle dynamics). (*E*–*J*) Quantification of clusters of encoding parameters: detection threshold, dynamic, and static firing (6 shown of 31 [*SI Appendix*, Tables 1 and 2]), averaged from four trials, from each neuron in each of the neuronal classes. * indicates statistically significant differences as empirically derived from hierarchical Bayesian model (stan_glm): 95% highest density intervals do not overlap. Ia: control (*n* = 11) and cOIN (*n* = 10); I_un_: control (*n* = 19) and cOIN (*n* = 5);_._ Ib: control (*n* = 14) and cOIN (*n* = 14); and II: control (*n* = 17) and cOIN (*n* = 14).

We found that errors in spike train encoding extended to all types of propriosensory neurons recorded from Apc^Pirc/+^+OX rats (cOIN) relative to Apc^WT^ (control) (Fig. 1 *A*–*D*). The detection threshold or lowest detectable level of muscle force by Ib neurons and for length changes by Ia, I_un_, and II neurons increased by two- to fivefold (Fig. 1 *E* and *H*). Firing responses to dynamic stimuli, specifically constant velocity stretch, were markedly reduced across all propriosensors (Fig. 1 *F* and *I* and *SI Appendix*, Table 1). Static muscle position also evoked abnormal firing that accommodated quickly and ceased prematurely in Ia, I_un_, II, and Ib neurons (Fig. 1 *G* and *J*). Altered dynamic and static firing was not simply attributable to increased detection threshold, since increasing background muscle length (L_o_ strain) by two- and threefold failed to restore neuronal signaling to control levels. These findings indicate that all muscle propriosensory neurons exhibited reduced sensitivity to dynamic and static features of muscle biomechanics as compared to control. Because each cell type provides unique sensory feedback essential for movement control (21, 23), these results suggest that chronic sensorimotor disabilities that follow cancer treatment depend on multimodal encoding defects shared across all muscle propriosensory cell types.

### Comparable Effects across Disparate Propriosensors.

**Fig. 2.**
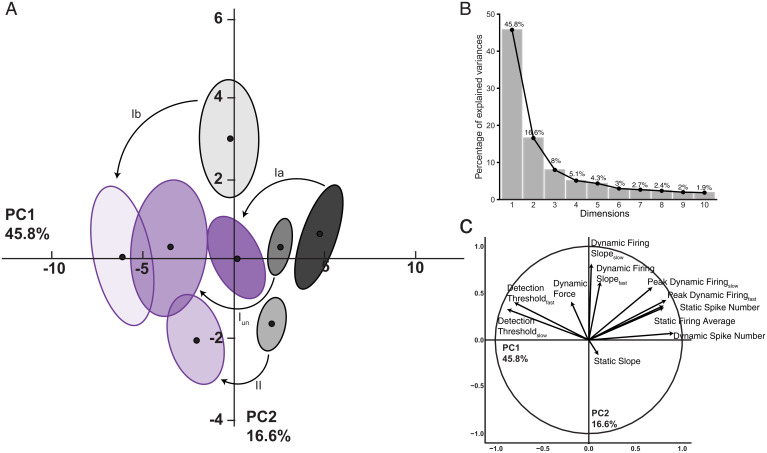
Consistent treatment effects emerge in latent encoding space for all propriosensors. PC analysis was applied to all (*n* = 31) encoding parameters measured in response to natural stimuli. (*A*) Neuron class means (average of PC1 to PC2 coordinates across all neurons in a given class and treatment group: black circles) are visualized in the new latent encoding space created by PC1 to PC2. Color-coded least-squares elliptical fitting (95% confidence) was computed to emphasize differences between neuron classes and experimental groups. Labeled arrows are drawn to indicate the magnitude and direction of treatment effects across neuron classes. (*B*) Scree plot indicating percentage of explained variance by each PC (gray bar, eigenvalue in percentage). (*C*) Vectors represent the magnitude and direction of correlation between individual parameters and the two latent features capturing the most variance (PC1 to PC2). Ia: control (*n* = 11) and cOIN (*n* = 10); I_un_: control (*n* = 19) and cOIN (*n* = 5);_._ Ib: control (*n* = 14) and cOIN (*n* = 14); and II: control (*n* = 17) and cOIN (*n* = 14).

We then characterized defects in neuronal signaling across all cell types in a common, stimulus-encoding subspace. We exploited principal component (PC) analysis for its potential to uncover latent patterns in the 31 measured and derived parameters of encoding to provide a parsimonious description of statistical features of interest [e.g., a common framework for comparing different cell types of proprioceptors and treatment effects (24)]. PC analysis identified a low–dimensional encoding space (PC1-2) in which the majority of observed treatment effects (62.4%) are conserved across all cell types (Fig. 2*A*). The scree plot (Fig. 2*B*) depicts the independent variance accounted for by each of the top 10 PCs. Cell-type means, 95% confidence ellipses, and arrows are drawn to indicate the magnitude and direction of treatment effects. All cell types shifted with cancer treatment leftward along PC1 by a similar magnitude. In addition, Ib neurons also displayed negative shifts along PC2. Fig. 2*C* displays the parameters highly associated with PC1 and PC2 (magnitude and direction of vectors) and indicates that increases in detection threshold and parallel decreases in static and dynamic firing rates are the defining constellation of cOIN effects that is conserved across neuron classes. Interestingly, while Ib neurons experienced shifts in PC1 space, they were the sole class of neurons associated with reduction in PC2 space. Three key inferences are drawn from this finding. First, reductions in PC2 space are dominated by less sensitivity to changes in force, a canonical feature of control Ib neurons. Second, cOIN Ib neurons adopt a “spindle-like” PC profile. Finally, distinct changes in PC space potentially reflect susceptibility to an underlying mechanism in addition to those observed in Ia, I_un_, and II neurons. Collectively, identification of a latent encoding space that is stable among different cell types suggests uniformity in cancer treatment effects that might derive from either damage to common ion channels [e.g., Kv3.3 (20)] or the continued expression of regulatory mechanisms that preserve relative differences among propriosensory phenotypes.

### Impaired Population Encoding.

**Fig. 3.**
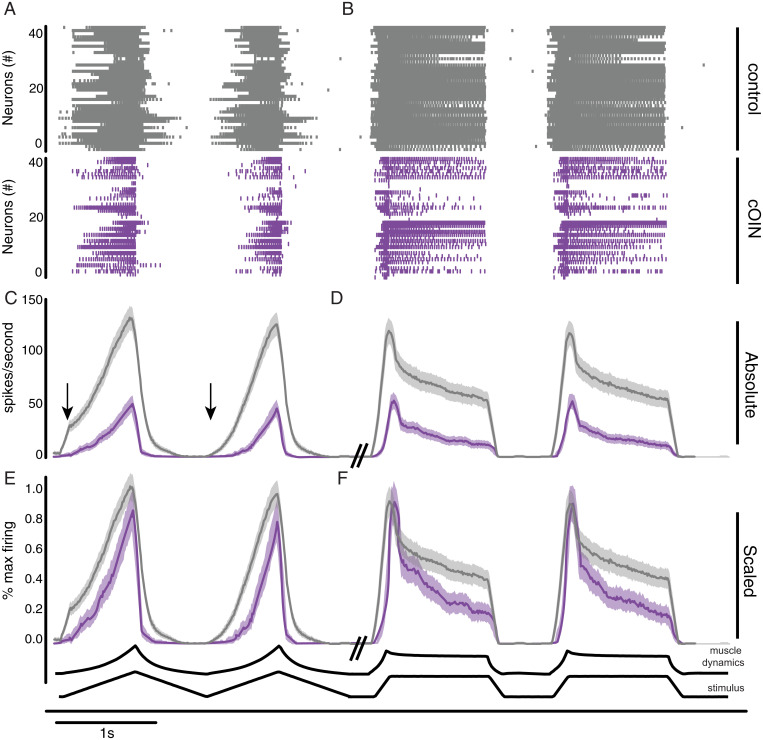
Chemotherapy for cancer impairs population encoding. Population code of 40 neurons is recorded from both control rats (gray) and cOIN rats (purple). Vertical lines in raster plot (*Top*) indicate the presence of individual spiking events during a 10-s representative recording as two slow (4 mm/s) ramp release (*A*), followed by two fast (20 mm/s) ramp-hold-release (*B*) mechanical stimuli. Average (*C* and *D*) and scaled (*E* and *F*) firing rate (solid lines) and SEM (shaded region) computed from the same recording epoch as above. Purple traces in *E* and *F* were scaled to the average maximum response magnitude, which among all types and multiple trials of stimuli was consistently obtained from ramp-hold-release stretch. (*C*) Arrows indicate the presence of history dependence in the first but not subsequent trials. Both population codes were constructed from the same distribution of neuronal classes: Ia (*n* = 10), I_un_ (*n* = 5), Ib (*n* = 14), and II (*n* = 11).

Next, we simulated the more naturalistic condition wherein muscle biomechanics were encoded by a multimodal population. For control (Fig. 3, gray) and cOIN rats (Fig. 3, purple), we analyzed an ensemble of spike trains compiled from 40 randomly sampled neurons. The average firing rate profile compiled from a population of propriosensors in cOIN rats displayed considerable differences from control in representing muscle biomechanics (Fig. 3 *C* and *D*). As expected, prominent deficits in the population code observed during slow ramp movements (Fig. 3*C*) include dynamic encoding (reduced gain in firing rate during ramp movement) and reduced history-dependent encoding (i.e., exaggerated response at onset of first movement) (Fig. 3*C*, arrows). The encoding of static position changes was truncated and sparse when present (Fig. 3*D*). Substantial delays in encoding movement onset were also observed during both slow and fast movement (Fig. 3 *C* and *D*). Expressions of blunted firing by the population represent losses in information that could not be attributed to simple scaling (Fig. 3 *E* and *F*).

### Chemotherapy for Cancer Impairs Cutaneous Sensory Encoding.

**Fig. 4.**
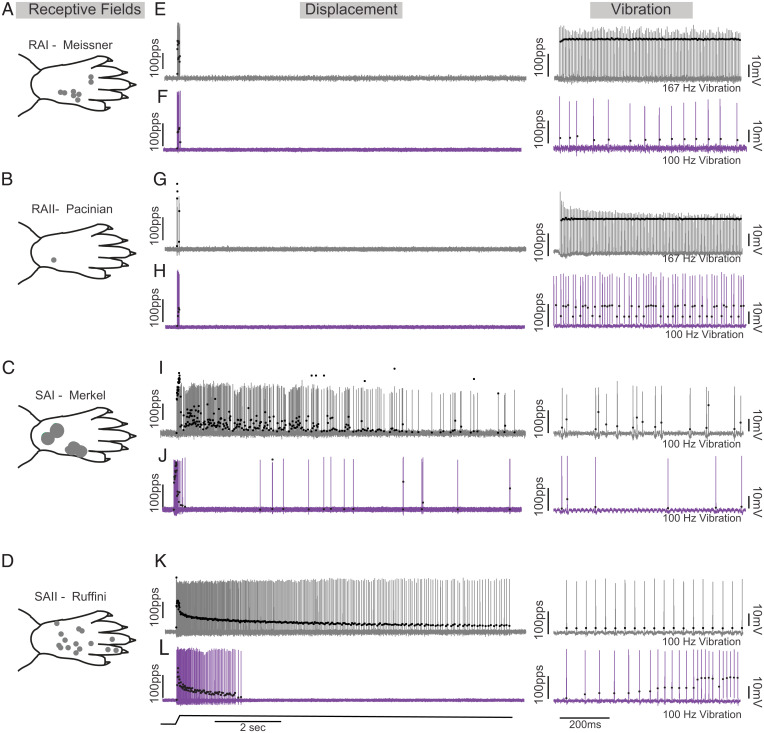
Chemotherapy for cancer impairs cutaneous sensory encoding. Representative cases of receptive fields (circles on paw traces in left column; *A*–*D*), spiking activity in control (gray; *E*, *G*, *I*, and *K*) and cOIN (purple; *F*, *H*, *J*, and *L*) as a measure of cutaneous sensory encoding in rapidly adapting Meissner corpuscles (RA; first row) and Pacinian corpuscles (PC; second row) and slowly adapting type I (SAI: Merkel corpuscles; third row) and type II (SAII: Ruffini endings; fourth row). Black circles plot instantaneous firing rates (pulses per second [pps]) of corresponding spike (action potential) intervals. The solid line below voltage recordings indicates the natural stimulation paradigm (i.e., displacement of the plantar skin in vivo utilized to study the cutaneous neurons [1 mm from resting length: L_o_]). Right columns of control (*E*, *G*, *I*, and *K*) and OX rats (*F*, *H*, *J*, and *L*) demonstrate representative responses to vibration stimulation. Note that control RAI and RAII neurons fire with high-fidelity to 167 Hz and 80-μm peak to peak amplitude displacements, whereas OX neurons fail to encode 100 Hz.

Cutaneous afferents also regulate coordinated movements by modulating the output of spinal motor circuits (25–28). To define the cell-type–specific contributions of altered cutaneous feedback to chronic movement impairments in cOIN, we recorded from rapidly adapting Meissner corpuscles (29) (RAI; Fig. 4*A*), Pacinian corpuscles (RAII; Fig. 4*B*), slowly adapting Merkel corpuscles (SAI; Fig. 4*C*), and Ruffini endings (SAII; Fig. 4*D*) while applying pressure to the plantar skin of the hindfoot in vivo, a different stimulating paradigm from muscle propriosensors. Stimulus encoding by cutaneous sensory neurons from control animals allowed clear discrimination among the four cell types (Fig. 4 *E*, *G*, *I*, and *K*; see *Materials and Methods*) and was consistent, along with their receptive fields (Fig. 4 *A*–*D*), with findings from previous studies (30). While SAI and II, RAI, and RAII neurons recorded from cOIN rats were also distinguishable based on their encoding characteristics (30), we observed cell-type–specific disruption in their canonical encoding phenotypes as compared to control (Fig. 4 *F*, *H*, *J*, and *L*). Stimulus discrimination (detection threshold) in SAII-type neurons was impaired (control: 4.96 ± 0.96 g, *n* = 7; cOIN: 13.59 ± 1.52 g, *n* = 13) while static encoding significantly decreased in both SAII and SAI neurons, changes associated with large reduction in capacity to sustain firing (control: 9.94 ± 0.013 s, *n* = 7; cOIN: 6.02 ± 0.791 s, *n* = 13). On first inspection, both RA-type neurons maintained several encoding characteristics (e.g., action potential generation in response to the onset and offset of stimuli). However, following chemotherapy, we observed that the proportion of RAII- (*n* = 3/3 in control and *n* = 2/10 cOIN) and RAI- (*n* = 22/30 in control and *n* = 3/15 cOIN) type neurons that respond with at least 50 pps to high-frequency vibration was reduced (Fig. 4 *F*, *H*, *J*, and *L*). Unexpectedly, RAI-encoding threshold dropped by 45.7% (control: 18.9 ± 4.63 g, *n* = 39; cOIN: 10.26 ± 3.37 g, *n* = 33), rendering them more sensitive to skin displacement, yet their overall peak dynamic encoding frequencies were significantly lower. These data demonstrate that defective encoding of vibration by RA neurons offers a mechanistic explanation for reduced sensitivity to skin vibration observed clinically. Collectively, we find substantial and often heterogeneous alterations among the cutaneous sensory–encoding modalities that provide plausible mechanistic explanations of the diverse sensory symptoms, touch as well as proprioceptive, experienced by patients, independent of degeneration.

### Impairment within the Spinal Cord following Cancer Treatment.

**Fig. 5.**
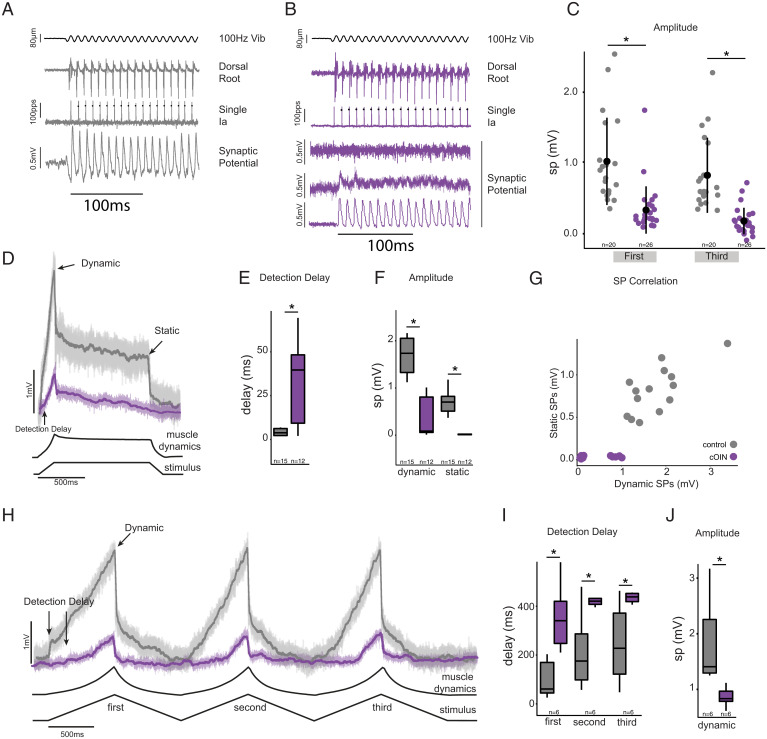
Synaptic potentials in sensorimotor circuit blunted after cancer treatment. Intracellular recordings in motoneurons are illustrating the representative amplitude and time course of excitation-dominated synaptic potentials (SP) evoked by muscle stretch. Responses to low-amplitude (80-μm peak to peak) 100 Hz muscle vibration (Vib) selectively activates Ia neurons in control (*A*) and cOIN rats (*B*). Representative intracellular records from Ia neuron and composite dorsal root (average of L5). Tight coupling exists between sensory input and synaptic potentials in control whereas cOIN synaptic potentials demonstrate three patterns (from three different motoneurons): no detectable potential (*Top*), decaying (*Middle*), and present (*Bottom*). (*C*) Quantification of synaptic potential peak amplitude for the first and third oscillation during vibration. Two muscle perturbations (solid lines) are the following: ramp-hold-release (*D*) and three-ramp release (*G* in control [gray] and cOIN rats [purple]). Color-coded solid lines and matching transparent backgrounds indicate mean and variance of voltage deviation recorded at ≤−60 mV, in which upward trajectories represent depolarization. Arrows in *D* and *H* indicate synaptic potentials regions for subsequent quantification: detection delay, dynamic peak (peak depolarization at maximum perturbation amplitude), and static (steady-state depolarization at end of hold phase of stretch). (*E*, *F*, *I*, and *J*) Quantification of synaptic potential regions of interest. (*G*) Plot of static versus dynamic synaptic potentials in both experimental groups. Note the systematic reduction in dynamic information decoding and near absence of any static information decoding in cOIN rats. * indicates statistically significant differences as empirically derived from hierarchical Bayesian model (stan_glm): 95% highest-density intervals do not overlap. Control (*n* = 12) and cOIN (*n* = 25).

Next, we examined the integration of propriosensory signals within central nervous system elements of the sensorimotor circuit. Synaptic potentials were recorded intracellularly from spinal motoneurons in response to the same forms of muscle stretch applied above in studying muscle propriosensors (Fig. 5). In control rats, synaptic potential waveforms, predominantly depolarizing excitatory, resembled the profile of muscle dynamics responding to different rates and positions of muscle stretch. By comparison, synaptic potentials in cOIN rats were delayed, blunted, and distorted (Fig. 5). For example, identical ramp-hold-release muscle stretches evoked synaptic potentials (Fig. 5*D*), which, in comparison with control, were delayed (Fig. 5*E*), 4.5-fold smaller in peak dynamic amplitude, and 35-fold smaller in static amplitude in cOIN rats (Fig. 5 *F* and *G*). In response to slower muscle stretch, synaptic potential onset was delayed from control by 100’s of milliseconds (Fig. 5 *H* and *I*).

While synaptic potential deterioration under the conditions just described demonstrates dysfunction expressed by central elements of the circuit, it does not necessarily indicate central origins. Synaptic potential decay originates at least partially in the periphery from defective firing of propriosensors. In order to test for possible central defects, we employed muscle vibration to evoke sensory firing that is unaffected by cancer treatment. In contrast with impaired firing responses to other forms of muscle perturbation (Fig. 1), vibration in cOIN rats selectively evokes Ia firing that retains its normal high-fidelity encoding, spiking with each and every high-frequency vibratory oscillation in muscle length [Fig. 5 *A* and *B*; see also (20)]. Reliable Ia neuron activation was verified in cOIN rats by observing stable firing by the population of responding Ia neurons recorded extracellularly in dorsal roots while synaptic potentials were being recorded from motoneurons (Fig. 5 *A* and *B*) Despite confirmed regularity in Ia neuron spike trains evoked by muscle vibration, motoneurons in cOIN rats responded with synaptic potentials that were either undetectable or unsustained (Fig. 5*B*), in sharp distinction with the discrete and sustained synaptic potentials that were readily and almost invariably observed in control rats. In comparison with control, vibration-evoked synaptic potentials in cOIN rats were reduced in amplitude (Fig. 5*C*) and expressed in a significantly smaller proportion of motoneurons (*n* = 7/26 versus 19/20, respectively), posterior probability distributions indicate a 37% (95% highest density interval [HDI]: 22 to 54%) versus, 92% (95% HDI: 80 to 99%) proportion of detectable synaptic potentials in cOIN and control. These findings definitively establish functional impairment localized within the spinal cord, independent of defects in the circuit’s peripheral functions.

### Circuit Defects Predict Kinematic Errors.

**Fig. 6.**
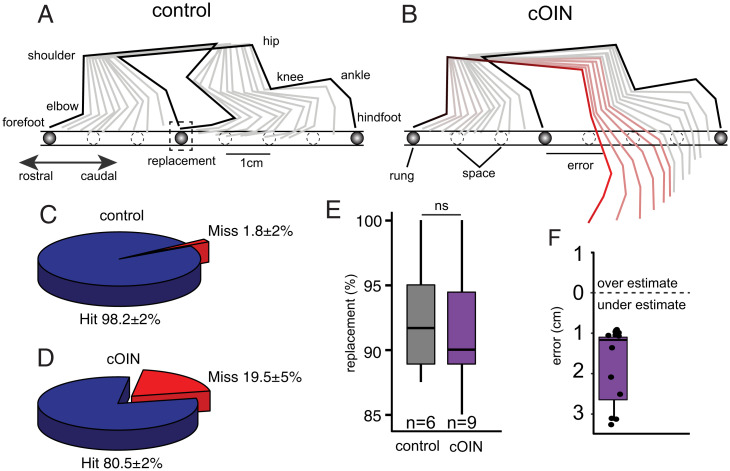
Movement disorders induced by cancer treatment predicted by circuit dysfunction. Labeled stick diagram of fore- and hindlimb joints. Stick figure decomposition of fore- and hindlimb movement during single step for a representative control (*A*) and cOIN rat (*B*) during crossing of an elevated downslope ladder with rungs (solid) unevenly and unpredictably spaced. (*C* and *D*) Pie charts summarize total percentage of hindfoot hits and misses for each experimental group. (*E*) Bar graph quantifying proportion of hindfoot replacements (illustrated in dotted box in *A*) in both control (gray) and cOIN rats (purple). Percent replacement analysis was restricted to steps in which cOIN and control rats successfully placed a hindlimb on a rung (i.e., a successful hit). (*F*) Quantification of magnitude and direction of initial hindfoot error with respect to intended target. ns indicates not statistically different as empirically derived from hierarchical Bayesian model (stan_glm): 95% highest-density intervals overlap.

We performed high-resolution video recordings to evaluate sensorimotor performance during a precision movement task [i.e., ladder rung walking (10° downslope)]. All control rats progressed across the ladder with minimal errors (1.8 ± 2%, *n* = 6 rats). Quantification of fore- and hindfoot positioning revealed that control rats targeted rungs precisely and consistently adopted identical hindfoot placement strategies, such that the hindfoot was subsequently placed at the same location as the ipsilateral forefoot as the rat traversed the ladder (Fig. 6 *A* and *E*, dashed box and Video 1). We utilized this conserved motor control strategy, called “replacement,” to define the forefoot as a predictor (“reporter”) of the intended target for the ipsilateral hindfoot placement (Fig. 6 *A* and *E*). With knowledge of the intended movement goal of the hindlimbs, we quantified the magnitude and direction of sagittal plane errors that occurred during ladder rung walking.

cOIN rats demonstrated significantly more errors in precise hindfoot placement (Fig. 1*D*), as previously reported (20), yet when they successfully placed a hindlimb, they achieved >90% replacement, demonstrating a conserved forefoot reporter strategy (Fig. 1*E*). Using the forefoot as a reporter, we then quantified the magnitude and direction of hindfoot errors. Surprisingly, all initial errors undershot the intended target (Fig. 6*F*, 1.72 ± 0.61 cm, *n* = 11). Undershooting targets (e.g., hypometria) has been observed in other nervous system lesions (31). It is hypothesized to represent a compensatory strategy to simplify (e.g., decompose) dynamic control over a limb by reducing the need to dynamically account for interaction torques occurring at the moving joint (31). Furthermore, cOIN rats demonstrated a greater splayed foot pattern of support, a feature that is consistent with a conservative gait pattern employed to reduce instability. Altered precision grasp behavior (*SI Appendix*, Fig. S1) might also be explained by corrupt cutaneous feedback discovered here that is consistent with precision grasp defects induced when cutaneous feedback circuits are genetically disrupted (32). These findings corroborate and extend our earlier demonstrations of movement disorders induced in cOIN rats (20, 33) and set the context required for testing association with sensorimotor-encoding defects.

## Discussion

Our study identified functional damage caused by cancer treatment through compounding factors acting at multiple sites in a network of neurons responsible for sensory-guided movements. In a rat model of cOIN, we assessed information transfer in vivo through a spinal sensorimotor circuit that normally acts to coordinate muscle responses to perturbations in movement and posture. Here, we show circuit dysfunction expressed both peripherally in sensory encoding by a diverse population of propriosensors and centrally in the transmission of propriosensory signals through neural pathways converging on motoneurons. As neural signals moved through the circuit, sequential errors compounded to the point that motoneurons, the final common pathway in neural instruction for movement, responded to propriosensory signals with synaptic potentials that were severely blunted and distorted. Spinal circuit dysfunction was consequential in predicting significant errors in propriosensory-guided movement behaviors demonstrated here in our rat model and reported for people with chemotherapy-induced neuropathology. We conclude that sensorimotor disability induced by cancer treatment resulted from multiple functional impairments compounded across peripheral and central components of spinal sensorimotor circuits.

### Propriosensory Translation in a Spinal Sensorimotor Circuit.

**Fig. 7.**
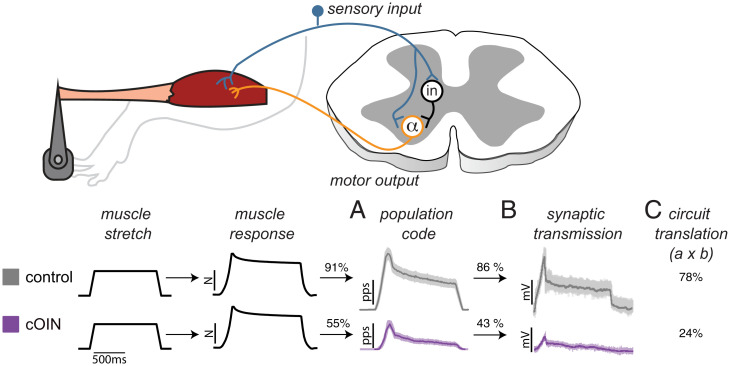
Compounding sensorimotor circuit dysfunction in cOIN. Diagram shows key stages in translation of propriosensory signals in a spinal circuit: Propriosensory neurons (blue object) encode and conduct signals from the periphery into the spinal cord, where synaptic potentials are produced in α motoneurons (orange object) either mono- or polysynaptically through interneurons (in). Records linked to corresponding recording sites in the circuit are displayed in temporal sequence from left to right for both control and cOIN rats. Imposed muscle stretch (ramp-hold-release change in length) and resultant muscle dynamics (viscoelastic restoring force in nonreflexive muscle) indistinguishable between control and cOIN rats. Firing elicited in different propriosensors (Ia, I_un_, II, and Ib) recorded from dorsal roots was compiled as a population code (cf Fig. 3) embodying all neural processes in the peripheral component of the circuit. As a result of its reduction, the population code’s representation of muscle dynamics fell from 91 to 55% in accuracy, quantified as percent variance accounted for (R^2^). In the final circuit stage examined here, motoneuron synaptic potentials degraded in cOIN relative to control, reducing their accuracy in representing the population code in half from 86 to 43%. In this comparison, group accuracy would have been identical if circuit impairment was restricted to the periphery. When compounded as the product of percent accuracy of independent peripheral (*A*) and central (*B*) processes, overall accuracy in circuit representation of muscle dynamics dropped from 78% in control to 24% in cOIN (*C*).

Fig. 7 illustrates sequential physiological responses recorded in vivo from an intact sensorimotor circuit reacting to naturalistic stretch of a nonreflexive muscle. Beginning in the periphery, the muscle’s dynamic response to stretch was represented by a neural population code constructed from the ensemble firing of multiple propriosensory neurons and classes. The accuracy in population code representation of muscle dynamics, measured as percent variance accounted for, achieved 91% in control rats (Fig. 7*A*). Next, in sequence, the population code entered the spinal cord, where it translated through converging neural pathways to produce synaptic potentials in motoneurons. In this central portion of the circuit, synaptic potentials represented the population code with 86% accuracy in control rats (Fig. 7*B*). In combination, the product of these independent peripheral versus central processes yielded 78% accuracy in the circuit representation of muscle dynamics (Fig. 7*C*). By contrast, the same measure fell to 24% accuracy in cOIN as a result of peripheral and central impairments discussed in *Impairment in the Motor Portion of the Circuit* (Fig. 7*C*).

### Circuit Impairments in the Periphery.

Comparison of population codes in Fig. 7 reveals obvious decay in cOIN rats and a drop in accurate representation of muscle dynamics from 91 to 55%. Deterioration of the population code (Fig. 5) emerged from robust encoding defects shared by all types of muscle propriosensors (Figs. 3 and 4). Substantial changes observed here for Ib and II neurons in cOIN rats contrast with the much weaker or absent effects we found in an earlier study of these neuron types in healthy rats treated with oxaliplatin (33). This difference is consistent with our finding, previously demonstrated for Ia neurons only, that chronic neural deficits are exacerbated by systemic interactions between cancer and chemotherapy (20). In cOIN rats, robust misrepresentation of muscle dynamics by all muscle propriosensors left none to compensate for diminished signaling by others [cf. (34)].

Motivated by reports of their capacity to compensate for reduced signaling in muscle propriosensors (35), we examined cutaneous sensory neurons in cOIN rats. Defective firing responses representing dynamic and static stimulus features of skin pressure were variously expressed by classes of rapidly and slowly adapting cutaneous mechanosensors. These findings show that cancer treatment impairs two major somatosensory modalities, proprioception and touch, thereby diminishing chances for compensation. Encoding errors broadly distributed by mechanosensors variously diverging and converging in numerous neural circuits, both spinal and supraspinal, would necessarily and severely impair sensorimotor behaviors and distort movement perception. Through their origins in neural signaling defects, proprioceptive and touch disabilities fall in a category with pain disorders, which are thought to result in part from modification of central neural circuits secondary to aberrant signaling (36).

Information about internal muscle dynamics and cutaneous pressure cannot be substituted by other sensory modalities [e.g., vision and vestibular (37, 38)]. While vision plays a critical role in assisting movement, it provides information only for body parts within the field of vision and only about the kinematics but not the kinetics of movement. While the vestibular system encodes kinematics and kinetics of head movements in the gravitational field, it provides essentially no sensory information about limb movements. As a result, losses of propriosensory signals would necessarily result in disabilities entirely consistent with those observed after cancer treatment.

### Circuit Impairments in the Central Nervous System.

If cancer treatment produced no damage in central circuit elements, then synaptic potential representation of the population code should have approximated the 86% accuracy observed in control rats. Accuracy was instead cut by half to 43% (Fig. 7), signifying expression of an effect isolated within intraspinal circuit elements. The drop in accuracy would not necessarily indicate central dysfunction because spinal circuits may normally respond to a decrease in population code with a disproportionately larger decline in synaptic potentials. This possibility requires no central defect. However, we found for control rats that synaptic potentials and the magnitude of muscle stretch decrease proportionally (*SI Appendix*, Fig. S2). That finding shifted attention to the possibility that cancer treatment impairs neural integration at some site(s) within the spinal cord. This possibility was definitively demonstrated in finding that muscle vibration, despite activating Ia neurons in cOIN rats as it does in controls, fails completely or partially to evoke synaptic potentials in motoneurons as it does reliably in control rats (Fig. 5).

The central defect responsible for diminishing synaptic potentials evoked by muscle vibration was not resolved in the present study. However, ample documentation of intraspinal connections between Ia neurons and motoneurons limits candidate mechanisms. In healthy animals, muscle vibration selectively activates Ia neurons to produce monosynaptic potentials in motoneurons (39). Failure in generating Ia synaptic potentials in motoneurons involves one or more of three processes. Least likely of the three is damage to the motoneurons’ electrotonic properties, which are unchanged in cOIN rats (12, 19) and adequate to support nominal synaptic potentials responding to other stimuli, ramp stretches, for example. Alternatively, Ia synaptic potentials may be impeded by a breakdown in action potential conduction through the last 3 mm of the Ia neuron’s axonal intraspinal projection to motoneurons, possibly by an abnormal conduction block occurring during vibration’s high-frequency activation. While the latter possibility is not ruled out by findings presented here, some doubt arises from the normal capacity of Ia axons to conduct action potentials at high frequencies over ca. 95% of their length from the sensory receptor up to the dorsal root recording site. A third possibility is that cancer treatment impairs transmission at Ia–motoneuron synapses. The rapid decay of synaptic potentials we observe in cOIN rats (Fig. 5*B*) may reflect pathological exaggeration of synaptic depression, a process which is well documented in other systems and attributed to activity-dependent processes in synaptic transmission. The plausibility of synaptic dysfunction gains support from one earlier study that demonstrates modification of activity-dependent plasticity at synapses in a spinal pain circuit responding to acute administration of oxaliplatin in mice (40). While recognizing the need for definitive demonstration, we propose that defects in synaptic transmission, whether effected pre- or postsynaptically, contribute to the impaired translation of propriosensory signals that we find within the central nervous system of cOIN rats.

### Impairment in the Motor Portion of the Circuit.

Spinal sensorimotor circuit operation continues in sequence as motoneurons integrate propriosensory synaptic potentials into spike trains that control muscle contraction. Recently, we reported that motoneuron firing is irregular and unregulated in cOIN rats as a result of biophysical changes intrinsic to the motoneurons’ intraspinal, encoding elements (12). Those findings support two conclusions relevant to the present report. First, dysfunction localized to the motoneurons’s central encoding region provides further evidence that cancer treatment impairs processes residing within the central nervous system. We now know that cancer treatment induces damage to two fundamental processes in circuit function, namely neuronal excitability and circuit operations. Second, motoneurons represent yet another independent source of impairment that would compound with others to drive circuit representation of propriosensory signals further below 24% accuracy (cf. Fig. 7). With respect to the final expression of circuit function, namely muscle force generation, two further factors require consideration. Skeletal muscle wasting (cachexia), together with alteration of contractile elements, with cancer treatment certainly influences the motor response to propriosensory signaling (41). The neuromuscular junction is also disrupted acutely by chemotherapy, but the chronic status of transmission at that synapse remains unstudied.

### Functional and Clinical Consequences of Propriosensory-Guided Movements.

Defects in spinal sensorimotor circuits were consistent with behavioral disability measured in the same rats. Propriosensory signaling is required for success in walking downhill on unevenly spaced ladder rungs, especially for hindlimb placement that occurs outside the rat’s field of vision. Nearly flawless task execution by control rats contrasted with cOIN rats in which steps were interrupted by errors, always initial undershoots in hindfoot placement on the intended rung (Fig. 6*B*). The undershoot fits well with spinal sensorimotor circuit dysfunction identified in these same cOIN rats. When extensor muscles at the ankle, knee, and hip were stretched as the hindlimb swung forward, propriosensory activation would have been delayed, reduced, and foreshortened with the likely biomechanical consequence of producing the undershoot. This supposition is supported by knowledge that excitatory synaptic drive from propriosensors contributes substantially to recruiting muscles during walking (15, 23, 25). Further support derives from similar disability during ladder walking by mice with genetic knock down of muscle spindles (34). Phenotypic similarity in these two conditions may seem at first surprising given that propriosensory signaling by muscle spindles is largely eliminated in Egr3-knockout mice (34, 42) but partially retained by muscle spindle propriosensors in cOIN rats (Fig. 1). However, our findings show how the addition of downstream defects in the spinal sensorimotor circuit function would effectively disable propriosensory contributions to movement control.

Months and years following treatment with a variety of chemotherapy agents, people report a range of signs and symptoms of diminished sensorimotor function and mobility including the following: ataxia, susceptibility to falls, and clumsiness (43–46). Formal study of sensorimotor dysfunction has focused on postural instability. During quiet standing on force plates, the center of pressure excursions is larger and faster, and corrections are delayed relative to healthy controls (18). These aberrations are predictable from reduced and delayed signaling initiated by faulty propriosensory encoding (Fig. 3) that carries through central sensorimotor circuits. Sensory detection delays observed in this study exceeded 100 ms during slow movements at low frequencies comparable to those that dominate sway during standing balance in humans (0.1 to 1 Hz) (47). In these cases, response delays exceeding 100 ms would lead to a critical level of instability in quiet standing (43, 48, 49). The overall reduction in propriosensory signal intensity to less than half normal would diminish synaptic drive to neural networks. These propriosensory deficits, uncorrected and exacerbated by impaired integrative functions within the central nervous system, would necessarily and severely hinder motor responses, as well as sensory perceptions during unexpected perturbations in posture and gait (34, 38, 44, 45, 50–52).

While chronic sensorimotor disability following cancer treatment may be attributed to errors in propriosensory signaling as just described, disability is commonly explained by structural degeneration of peripheral nerves. There are, however, two distinctions that might prove useful to clinical diagnosis. First, the response delays in propriosensory signaling associated with defective encoding, but generally not with axon degeneration (53), could assist differential diagnosis depending on the occurrence of movement delays. Second, in the reasonable assumption that encoding errors extend to muscles throughout the body, proprioceptive disorders in proximal limb movements would be assignable to functional neuropathy rather than to axon degeneration that concentrates distally in limbs (17).

The clear message from present results, gathered with earlier findings, is that cancer treatment produces the persistent expression of dysfunction at multiple sites, both peripheral and central in spinal sensorimotor circuits. Each site alone has the capacity to interfere with propriosensory-guided movements and postures. Although ongoing study suggests that the magnitude of separate defects may vary among individual animals, we show here how compounding full and simultaneous expression of impairment at all sites would drive sensorimotor circuits into functional collapse. These observations impact development of effective treatments not yet available for restoring normal propriosensory behaviors postcancer treatment.

## Materials and Methods

### Animals and Experimental Groups.

All procedures and experiments were approved by the Georgia Institute of Technology Institutional Animal Care and Use Committee. Adult (250 to 350 g) female and male Fisher 344 (F344) (*Apc^WT^*) rats and rats carrying a germline *Apc gene* mutation (*Apc^Pirc/+^*) (54) were studied. We adopted the *Apc^Pirc/+^* rat model of colorectal cancer developed in Fisher 344 (F344) (54) because it approximates human colorectal cancer with fully developed cancer at 4 mo of age. Unique characteristics of this experimental model have been previously described (20) and are outlined in *SI Appendix*, *Supplemental Methods*.

All animals were housed in clean cages and provided food and water ad libitum in a temperature- and light-controlled environment. *Apc^WT^*+control and *Apc^Pirc^*^/+^+OX rats are referred to as “control” and “cOIN” throughout this study. Through this comparison, we evaluated the joint effects of cancer and OX on the sensorimotor circuit, a comparison most relevant to clinical translation.

### In Vivo Procedures.

All treatments and in vivo procedures have been previously described (20, 21, 55–58). Briefly, 5 wk after achieving clinically relevant chemotherapy doses, rats were deeply anesthetized by inhalation of isoflurane (5% in 100% O_2_) and for the remainder of the experiment via a tracheal cannula (1.5 to 2.5% in 100% O_2_). Vital signs were continuously monitored, including core temperature (36 to 38 °C), PCO_2_ (3 to 5%), respiratory rate (40 to 60 breaths/min), pulse rate (300 to 450 bpm), and SPO_2_ (>90%), to ensure anesthesia and overall animal health. Lumbar dorsal roots, muscles, and nerves in the left hindlimb were surgically exposed and prepared for stimulation and recording, as previously described (20, 21).

To record from cutaneous afferents, this outlined surgical procedure was used, deviating only that the posttibial nerve was isolated and put in continuity with a bipolar-stimulating cuff electrode; all other nerves were crushed. Calibrated von Frey filaments were used on the glabrous skin of the left hindlimb (bottom of the foot) to identify the force threshold and receptive field of cutaneous afferents. Using a servomotor with a wooden dowel (diameter of 2 mm) attached to a lever arm, a ramp hold release (2 mm, 20 mm/s, 10 s hold) was used to constantly displace the skin of the receptive field. In addition, 100 Hz vibration (80 µm amplitude) was applied to further discern the identity of cutaneous afferent.

### Statistical Analysis.

PC provided unsupervised dimensionality reduction for the multiple features of neuron signaling, in an attempt to identify dominant patterns of covariation across neurons and treatments (24). PC analysis and visualization was performed with the *factoextra* (59) and *FactoMineR* (60) in the R environment (version 3.5.0) (61).

We compute the posterior distribution for the difference in synaptic potential detection rates between the two groups. For this model, we suppose that, in each group, there is a probability of having a synaptic potential being detected called θ and that the counts we see result from a binomial process. We fit two models using Beta distribution (generates values between 0 and 1) as priors. The first model utilized a flat, uninformative prior by using a = 1, b = 1 [i.e., Beta (1, 1)]. This prior considers all probabilities from 0 to 1 as equally plausible. We then utilized a Beta (5, 1) to incorporate our expectation that a large fraction (if not all) of motoneurons have detectable synaptic potentials in response to muscle vibration (39). We report the more conservative Beta (5, 1) results in the present study. All other statistical techniques for evaluating neuronal encoding have been described in previously published reports from this laboratory (20, 62). All models were developed with the *rstanarm* package (version 2.18.1) (63) in R (version 3.5.0) (61). Models were validated by computing out-of-sample predictive accuracy using Pareto-smoothed importance sampling (64) to perform leave-one-out cross-validation, as previously described (12, 20). Summary statistics of observed data are reported as mean ± SD.

### Population Code Construction.

Intracellular recordings from physiologically identified sensory neurons of all four classes were used to construct population codes (ensembles of neuronal firing). Since identical natural stimulation patterns were used for all neuronal recordings, it was possible to precisely align the spike trains recorded independently from all neurons with the stimulus profiles. This allowed us to model population codes of physiologically identified neurons of precisely known locations from control and cOIN animals. Populations were constructed from the same distribution of neuronal classes: 10 Ia, 5 I_un_, 14 Ib, and 11 II. Data are presented as absolute and scaled coding averages, in which scaling procedures normalized data to have values between 0 and 1.

### Synaptic Recordings from Motoneurons.

Synaptic potentials were recorded intracellularly from triceps surae motoneurons in response to the same, mechanical, biomechanical stimuli of the triceps surae muscles utilized for studies of sensory neurons as described previously (65). Movements were repeated for 15 to 20 trials. Synaptic potentials were then averaged on stimulus onset.

### Behavioral Analysis.

We used the ladder rung–walking task as a validated outcome to detect and describe sensorimotor deficits as previously described (20, 66). Kinematic errors (sagittal plane) in hindfoot placement were measured as the distance from the intended ladder rung target. We determined the intended target of hindfoot placement by evaluating whether the hindfoot replaced the ipsilateral position of the forefoot during the step cycle (Fig. 6, dotted box). Analysis focused on initial hindfoot errors, excluding subsequent errors of hindlimbs and forefoot errors because adequate ground truths, could not be established (through the forefoot reporter strategy) to quantify kinematic errors.

Video recordings were analyzed on a computer equipped with DeepLabCut (version 2.1.5.2), a software based on deep learning to track user-defined body parts (67, 68). For hindlimb kinematics, the positions of the joints and toe tip were detected using DeepLabCut. The joint positions were used to extract the angles of the hip, knee, and ankle joints. Frames were excluded from the analysis if any limb joints or the toe tip had a likelihood of detection <0.8 by DeepLabCut. Frames were excluded from the analysis if any paws’ or joints’ speed exceeded 150 cm/s (i.e., the maximum locomotor speed).

## Supplementary Material

Supplementary File

## Data Availability

All data and code used are available in GitHub: https://github.com/nickh89/Neural_circuit_mechanisms_of_sensorimotor_disability_in_cancer_treatment. All other study data are included in the article and/or *SI Appendix*.
