# Supplementary material for: Neural circuit mechanisms of sensorimotor disability in cancer treatment
Source: Proc Natl Acad Sci U S A. 2021 Dec 15;118(51):e2100428118. doi: 10.1073/pnas.2100428118 (PMC8713769; doi:10.1073/pnas.2100428118)
Supplement: Supplementary File [file pnas.2100428118.sapp.pdf]

Supporting Information Appendix

Supplemental Methods

Classification of Muscle Afferents

Muscle afferents that fired during the rising phase of a twitch contraction of the triceps surae muscle when the triceps surae nerves were electrically stimulated were designated group Ib, while those that paused were classified as spindle afferents (Ia, I_{un}, II). Group II afferents are identified by a lack of 1-to-1 response to 80µm vibration at 100 Hz, and little to no history dependence when stretched by successive triplets of triangular stretches (3mm, 4mm/s). Both Ia and I_{un} have 1-to-1 fidelity to vibration and have a significant history dependence. When presented with ramp-hold-release stretches (3mm at 20mm/s, 1 second hold), Ia muscle afferents respond with an initial burst of high-frequency firing (>100pps) at the onset of muscle stretch. Muscle afferents that did not have an initial burst were identified as I_{un}.

Classification of Cutaneous Afferents

Slowly adapting type I (SAI: Merkel corpuscles; Fig 4c) respond to constant stimuli with high coefficient of variance (CoV) inconsistent firing pattern throughout the entire hold phase while type II (SAII: Ruffini endings; Fig 4d) fire regularly (low CoV) throughout the hold phase. Rapidly adapting Meissner corpuscles (RA; Fig 4a) and Pacinian corpuscles (PC; Fig 4b) respond with brief firing at the onset of the stimuli lasting no more than 2 seconds and respond 1-to-1 to vibration. Meissner corpuscles are differentially identified by an additional short burst of firing during the release phase of the stimuli.

Supplemental Figures

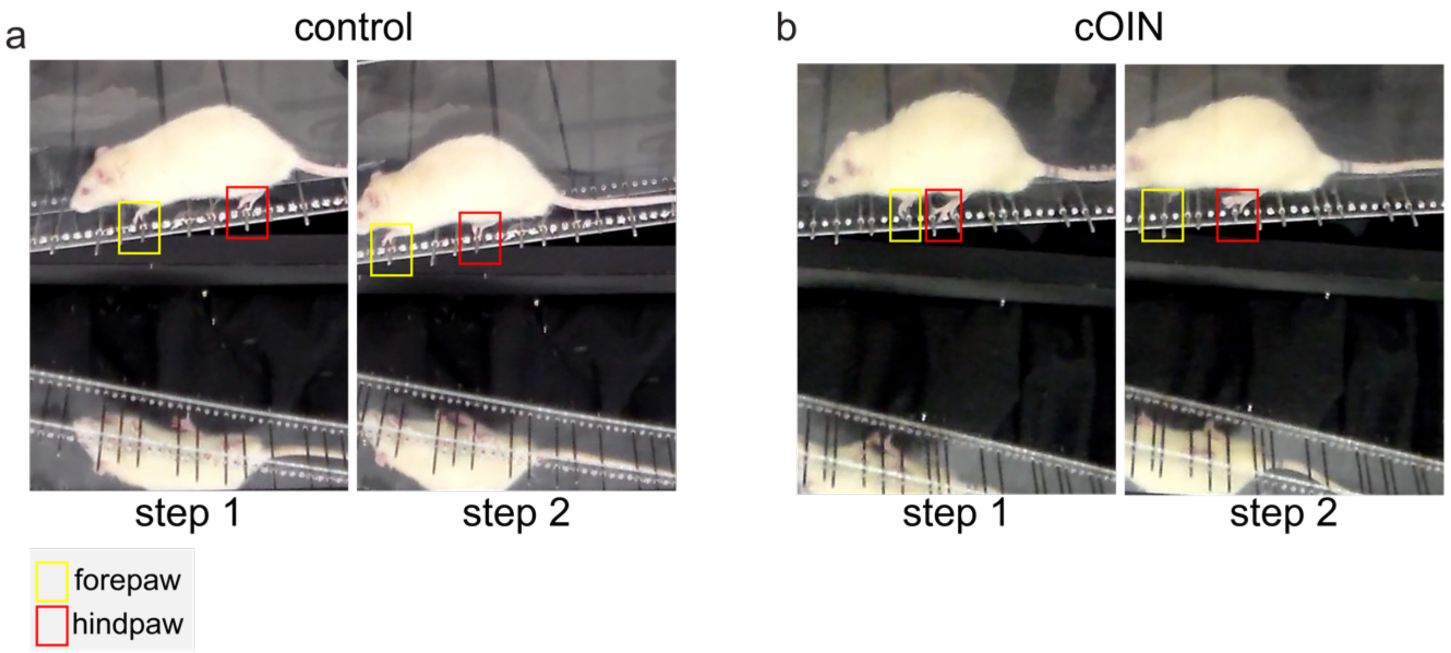

Figure S1. Grasp performance in skilled sensorimotor task altered by cancer treatment. Representative photographs show two fore- and hindpaw placements during skilled ladder rung walking with double image (simultaneous side and underneath views) of control (a) and cOIN (b). Fore- and hindpaw placements outlined in yellow and red respectively emphasize the fine motor skill (grasp) interaction between each paw and its associated rung. Note splayed paw strategy is adopted by cOIN rats in both the hind- and forepaws**.**

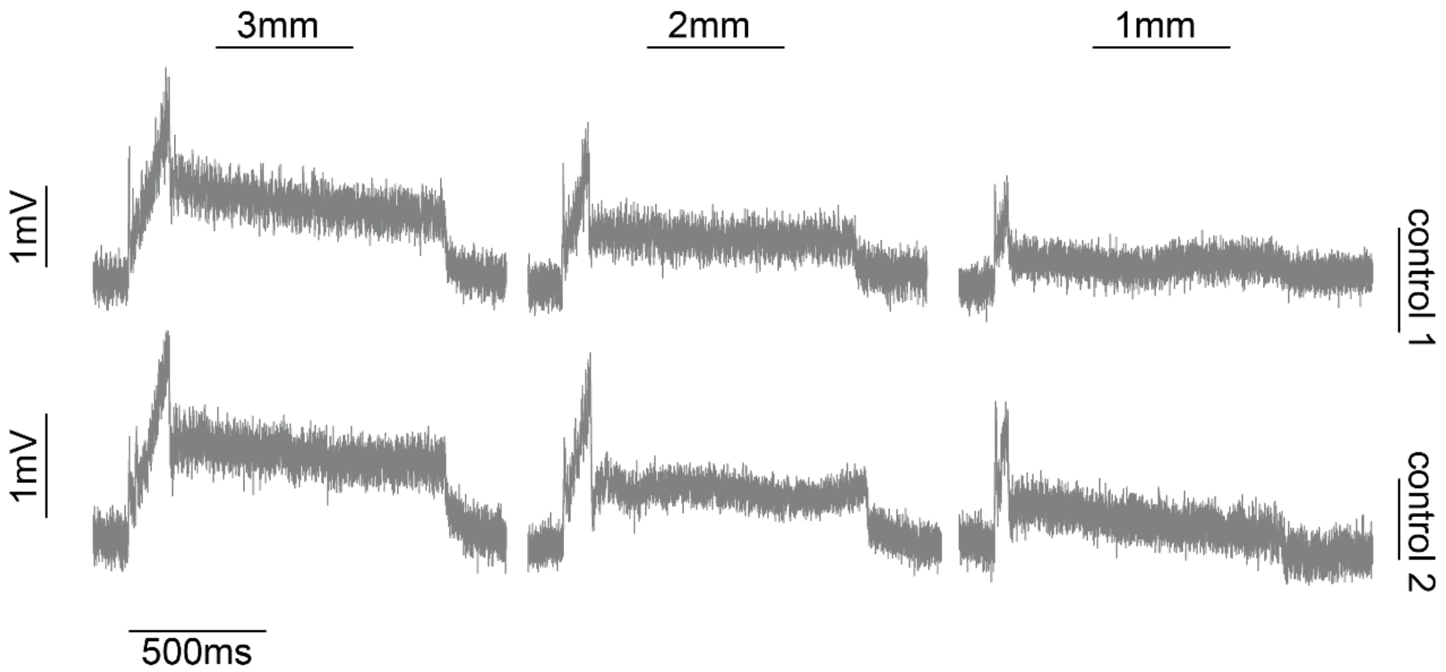

Figure S2. Linear effects of reducing stretch amplitude. Representative intracellular average synaptic potentials recorded in response to 3mm, 2mm, and 1mm stretch of the triceps surae muscle in two control rats.

Supplemental Tables

Functional Cluster	Definition
	Sensitivity/Threshold Functional Cluster
Thr.L3	Length at which 1st action potential is detected on the 3rd slow ramp (mm)
Thr.L	Length at which 1st action potential is detected on the fast ramp (mm)
Thr.T1	Time at which 1st action potential is detected on the 1st slow ramp (ms)
Thr.F3	Muscle tendon force at which 1st action potential is detected on the 3rd slow ramp (dN)
Thr.F1	Muscle tendon force at which 1st action potential is detected on the 1st slow ramp (dN)
Thr.T	Time at which 1st action potential is detected on the fast ramp (ms)
Thr.F	Muscle tendon force at which 1st action potential is detected on the fast ramp (dN)
Thr.L1	Length at which 1st action potential is detected on the 1st slow ramp (mm)
Thr.T3	Time at which 1st action potential is detected on the 3rd slow ramp (ms)
	Dyamic Signaling Functional Cluster
Dyn.slp1	Slope of instantenous firing rate during the 1st slow ramp
Dyn.pfr1	Peak firing rate achieved during the 1st slow ramp (Hz, pps)
Dyn.spkNum	Number of action potentials (spikes) during the fast ramp (#)
Dyn.DI	Difference between the peak dynamic firing rate and the static firing rate during half hold (Hz)
Dyn.spkNum3	Number of action potentials (spikes) during the 3rd slow ramp (#)
Dyn.F	Peak dynamic force
Dyn.slp3	Slope of instantenous firing rate during the 3rd slow ramp
Dyn.spkNum1	Number of action potentials (spikes) during the 1st slow ramp (#)
Dyn.IB	Peak instantaneous high-frequency firing rate achieved immediately after stimulus (Hz)
Dyn.slp	Slope of instantenous firing rate during the fast ramp
Dyn.IFRdrop	Drop in instantaneous firing rate after the release on the slow ramps (Hz, pps)
Dyn.pfr3	Peak firing rate achieved during the 3rd slow ramp (Hz, pps)
Dyn.pfr	Peak firing rate achieved during the fast ramp (Hz, pps)
	Static Signaling Functional Cluster
Stat.mSfr	Instantenous firing rate midway through the static hold (Hz, pps)
Stat.afr	Average firing rate during the static hold (Hz, pps)
Stat.HzSTD	The standard deviation of instantaneous firing rate during the static hold
Stat.LSpkF	The muscle force at which the last action potential occurs during the static hold (dN).
Stat.slp	Slope of instantenous firing rate during the static hold.
Stat.LspkT	The time at which the last action potential occurs during the static hold (ms).
Stat.HzEnd	Instantenous firing rate during at the end of the static hold (Hz)
Stat.spkNum	Number of action potentials (spikes) during the static hold (#)
	History Dependence Functional Cluster
HxDep.RDR	Difference between the number of action potentials (spikes) in the 1st and 3rd slow ramp

Supplemental Table 1: List of Neuronal Encoding Parameters Measured and derived parameters were computed offline. Letters in the left column indicate the specific parameters included in analyses for each functional feature cluster. Thr: threshold (9 parameters); Dyn: dynamic (13 parameters); Stat: static (8 parameters); HxDep: history-depen-dent (1 parameter).

	Group Iun				Group Ia				Group Ib				Group II			
	Control (n = 19)		cOIN (n = 5)		Control (n = 11)		cOIN (n = 10)		Control (n = 14)		cOIN (n = 14)		Control (n = 17)		cOIN (n = 14)	
Variable	mean	sd	mean	sd	mean	sd	mean	sd	mean	sd	mean	sd	mean	sd	mean	sd
Dyn.DI	106.721	34.664	84.375	31.222	120.228	42.871	108.851	37.617	68.414	26.731	26.990	31.755	61.284	19.312	38.342	21.432
Dyn.F	248.809	94.763	143.334	26.355	185.547	85.920	150.635	25.126	286.545	51.476	265.065	89.830	156.710	75.989	186.481	59.955
Dyn.IB	0.000	0.000	0.000	0.000	282.630	47.511	76.124	161.851	0.000	0.000	0.000	0.000	0.000	0.000	0.000	0.000
Dyn.IFRdrop	72.856	38.229	65.097	34.126	86.412	34.436	82.523	26.593	23.913	16.404	60.004	63.185	29.611	25.987	51.410	74.347
Dyn.pfr	173.788	32.362	103.325	30.795	203.160	52.362	134.274	13.306	145.776	50.533	40.781	43.925	127.929	22.048	72.083	36.717
Dyn.pfr1	131.930	19.846	73.429	29.210	150.517	39.368	107.618	24.640	149.606	43.729	53.725	35.024	102.294	33.539	64.590	24.136
Dyn.pfr3	134.156	30.467	61.897	41.942	137.506	53.580	99.154	19.367	136.603	41.454	41.594	37.309	95.995	36.802	54.436	27.127
Dyn.RDR	20.684	4.460	7.000	1.414	24.636	8.732	14.100	4.654	7.643	3.319	2.500	2.139	17.471	7.019	5.786	6.387
Dyn.slp	1459.368	613.347	1641.300	800.827	929.939	454.994	1373.122	559.972	2144.717	1210.637	336.439	514.782	700.222	293.248	777.857	527.275
Dyn.slp1	142.075	33.073	189.731	75.123	124.506	62.945	160.040	99.853	259.762	204.570	267.735	230.167	87.295	36.934	139.414	104.496
Dyn.slp3	213.729	54.577	225.448	155.631	206.581	104.425	250.559	107.541	377.859	119.671	202.510	183.990	147.042	77.572	135.779	103.031
Dyn.spkNum	13.118	2.634	4.650	1.167	18.765	4.681	9.900	2.846	7.982	3.148	2.000	1.724	11.544	2.210	5.232	2.773
Dyn.spkNum1	60.632	13.022	15.600	6.269	72.727	25.710	35.600	11.928	33.929	15.051	7.214	6.963	48.176	16.775	17.429	11.407
Dyn.spkNum3	39.421	11.544	8.600	6.542	48.091	18.075	21.500	7.920	26.286	13.708	4.786	5.177	30.941	12.372	11.643	6.464
Stat.afr	73.845	17.293	46.481	14.018	93.983	22.025	67.595	15.430	87.788	30.889	42.302	32.968	73.186	19.631	38.537	21.341
Stat.HzEnd	49.147	25.938	12.295	16.902	73.473	21.462	24.021	26.600	62.881	35.723	9.961	20.804	56.869	21.535	23.439	20.379
Stat.HzSTD	27.233	9.689	31.255	12.774	29.848	10.449	35.689	11.719	26.674	9.147	14.277	13.833	19.033	5.649	14.778	7.737
Stat.LSpkF	116.256	37.971	110.128	21.709	122.503	37.486	120.709	22.602	166.044	56.972	149.076	44.766	110.750	40.702	105.391	17.930
Stat.LSpkT	985.249	25.929	497.852	287.828	991.437	20.820	723.291	365.624	990.376	15.271	316.842	424.390	994.735	4.863	906.598	255.568
Stat.mSfr	65.265	22.808	19.925	17.720	82.929	20.830	34.777	23.414	75.542	31.507	13.056	24.453	66.645	19.956	32.913	20.899
Stat.slp	-58.453	24.069	-195.385	78.233	-59.424	26.099	-289.610	459.055	-61.811	19.554	-51.033	91.542	-44.305	10.340	-31.413	13.712
Stat.spkNum	64.386	19.589	16.533	14.569	85.636	21.791	36.800	23.270	79.946	32.037	14.125	25.074	67.985	20.613	33.393	21.055
Thr.F	17.587	6.942	41.439	22.570	9.857	3.541	20.620	12.152	47.862	38.890	96.394	73.011	11.025	3.446	31.605	30.085
Thr.F1	11.957	9.782	49.010	15.315	7.143	5.971	21.709	18.454	66.416	37.164	121.697	77.400	8.150	5.635	41.641	40.607
Thr.F3	31.059	11.770	78.010	41.566	19.442	10.939	37.722	14.722	83.461	34.873	126.476	92.659	17.647	12.343	47.445	37.408
Thr.L	0.506	0.345	1.378	0.729	0.090	0.037	0.648	0.490	1.010	0.690	1.859	1.042	0.159	0.118	0.887	0.815
Thr.L1	0.207	0.220	1.695	0.347	0.147	0.247	0.719	0.631	1.373	0.651	2.199	0.752	0.176	0.236	1.078	0.832
Thr.L3	1.008	0.380	2.332	0.418	0.839	0.406	1.482	0.347	1.780	0.365	2.135	0.967	0.759	0.428	1.443	0.579
Thr.T	25.018	16.882	112.062	112.570	6.184	4.503	33.012	24.487	50.712	34.332	96.963	53.453	8.841	5.585	45.318	39.988
Thr.T1	53.346	64.728	419.770	87.570	29.885	40.007	192.746	183.736	324.183	184.894	562.462	133.715	43.638	58.879	267.062	205.853
Thr.T3	271.172	80.483	585.552	105.378	217.566	101.107	393.542	116.038	457.515	90.747	605.407	140.057	200.447	100.272	367.204	142.586

Supplemental Table 2. Neuronal encoding parameters in control and cOIN. Parameters describing various aspects of sensory encoding (n=31) outlined along the vertical axis together with the cell-type specific quantification by experimental group distributed along the horizontal axis. Values are presented in mean±SD.
